# Deconvoluting gene and environment interactions to develop an “epigenetic score meter” of disease

**DOI:** 10.15252/emmm.202318208

**Published:** 2023-08-04

**Authors:** Alessio Butera, Lena Smirnova, Elisa Ferrando‐May, Thomas Hartung, Thomas Brunner, Marcel Leist, Ivano Amelio

**Affiliations:** ^1^ Chair for Systems Toxicology University of Konstanz Konstanz Germany; ^2^ Center for Alternatives to Animal Testing, Bloomberg School of Public Health Johns Hopkins University Baltimore MD USA; ^3^ Deutsches Krebsforschungszentrum (DKFZ) Heidelberg Germany; ^4^ University of Konstanz Konstanz Germany; ^5^ Chair for Evidence‐based Toxicology Johns Hopkins University Baltimore MD USA; ^6^ Chair for in Biochemical Pharmacology University of Konstanz Konstanz Germany; ^7^ Chair for in vitro Toxicology and Biomedicine, Inaugurated by the Doerenkamp‐Zbinden Foundation University of Konstanz Konstanz Germany

**Keywords:** Evolution & Ecology

## Abstract

Human health is determined both by genetics (G) and environment (E). This is clearly illustrated in groups of individuals who are exposed to the same environmental factor showing differential responses. A quantitative measure of the gene–environment interactions (GxE) effects has not been developed and in some instances, a clear consensus on the concept has not even been reached; for example, whether cancer is predominantly emerging from “bad luck” or “bad lifestyle” is still debated. In this article, we provide a panel of examples of GxE interaction as drivers of pathogenesis. We highlight how epigenetic regulations can represent a common connecting aspect of the molecular bases. Our argument converges on the concept that the GxE is recorded in the cellular epigenome, which might represent the key to deconvolute these multidimensional intricated layers of regulation. Developing a key to decode this epigenetic information would provide quantitative measures of disease risk. Analogously to the epigenetic clock introduced to estimate biological age, we provocatively propose the theoretical concept of an “epigenetic score‐meter” to estimate disease risk.

## Graphical abstract

In this commentary, I. Amelio and colleagues discuss the interplay between genes and the environment in relation to health and disease and propose a theoretical concept called the “epigenetic score meter” as a means for disease risk assessment.

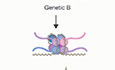

## Epigenetic records of gene–environment interactions

**Figure 1:**
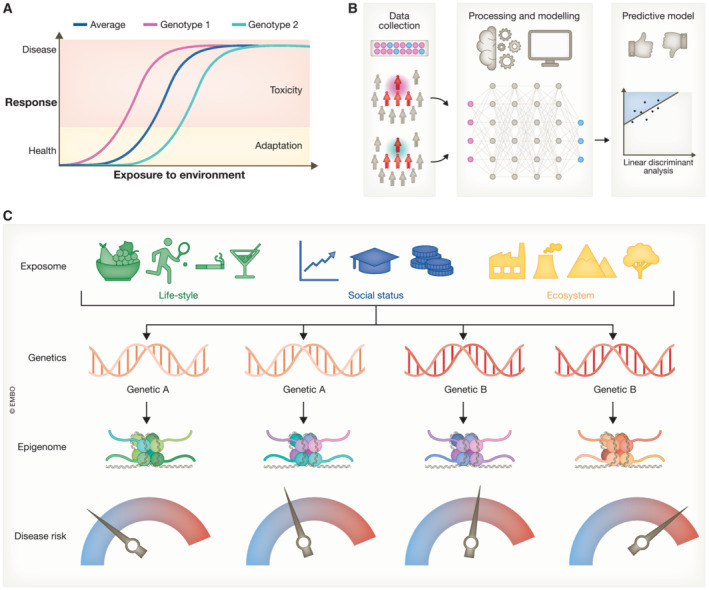
Epigenetic score meter of disease (A) Increased (dose and/or time) “exposure” to environmental factors determines a biological response that progressively shifts from health to disease. While most of the studies consider “average” population, stratification based on genetic background can allow identification of hyper‐ and hypo‐susceptible subgroups of individuals. (B) Mathematical modeling, supported by computational and/or machine learning approaches, has the potential to integrate population studies and develop predictive models of disease risks based on probabilistic assessment. We argue that a strategy of simplification (reduction of dimensionality) by using epigenetic information as integrated readout for the consequence of GxE might represent a successful route to achieve this goal. (C) Different categories of environmental factors, derived from lifestyle, social status, and the ecosystem, influence the disease risk. In a large fraction of cases, the interaction of these with genetics (GxE) determines the outcome for human health. Environment often accounts for multiple factors that interplay with each other, also in a timely fashion. Similarly, in the largest majority of cases, genetics involves genotypes produced by multiallelic variant patterns; thus, comprehensive deconvolution of GxE does not represent a feasible route. As both environmental factors and genetics can impinge epigenetic regulations, we argue that their interplay is recorded in the cellular epigenome and might represent a tool to quantitatively measure the risk of disease.

Genetics correlates with phenotypes, and differential responses to the same environments can be explained as different genetic makeups. Genetics can therefore stratify populations in high/low disease risk groups and instruct the regulators and policymakers on safety thresholds and measures (Fig 1A). The last decades have seen an exponentially increasing development of analytical tools (genomic approaches and omics technologies in general) that might assist delineation of boundaries and risk categories of the populations. The output of the huge amount of newly generated data do not linearly correlate with effective advancements in this area. Thus, despite being well‐accepted, the postulation of GxE as an explanation of biological phenotypes has remained of theoretical nature with a degree of provocative emphasis.

GxE interactions refer to the cooperative process in which biological phenotypes are shaped by contributions of these two categories of intrinsic (i.e., genes) and extrinsic (i.e., environment) factors. Applicable in theory to any biological system, this principle has important biomedical implications. Identification of the basis for differential susceptibilities and responses to environmental agents (genetic predisposition and nutrigenomics) or drugs (pharmacogenomics and toxicogenomics) has indeed important implications for public health and drug development. Historically, the concept was conceived as a simplistic dichotomy, in which genes (i.e., genetic traits) or environment (exposure, lifestyle, previous disease, and social experiences) were responsible for specific aspects of the interindividual variabilities. This view then expanded considering that additive and synergistic contributions of both genetics and environment participate in shaping a biological phenotype and providing an already complex scenario difficult to deconvolute with easily measurable parameters (Boyce *et al*, 2020). The most significant advancement in the complexity of this relationship is emerging with the apparent observation that the GxE interaction is often bidirectional and reciprocal with a multidimensional organization. Genes do not necessarily participate in the equation as fixed parameters, but regulation of their expression and function by environmental factors and stochastic events contributes to the response to concurrent and subsequent exposure. Moreover, concurrent regulation of multiple genes at different levels (from gene sequence to its transcription to the protein function) generates a multidimensional network of multilayered regulations often difficult to measure and record. Thus, the translation of the theory into practical applications has seen significant difficulties and the implementation is, in most cases, relatively far from reality.

A major example of controversy in this area is the debate on the “bad luck” cancer hypothesis. Here, arguments in support of the postulation that cancer is the result of mutations stochastically occurring during stem cell divisions are contraposed to arguments in support of significant contributions to cancer etiology derived from environmental exposure. These studies based on average populations were, however, overcome by more recent studies that were able to explain up to 50% of cancer with genetic predisposition (Ballinger *et al*, 2023). Clearly, the high degree of complexity with multiple levels of regulation limits the feasibility of the GxE deconvolution process. To approach such complex systems, we suggest undertaking a minimalistic strategy, using a single layer of control to exemplify the status of the GxE interaction.

Epigenetics refers to stable phenotypic changes that do not involve alterations in the DNA sequence, including chromatin state, and therefore the sum of modifications of DNA (DNA methylation) and posttranslational modifications of histones as well as non‐coding RNAs (ncRNA). It can be considered as the code of the functional status of a cell. Such a code incorporates information derived from the environment, the previous (social and disease) experiences, as well as the genetic makeup (polymorphisms and hereditary or sporadic mutations), which can have direct or indirect consequences for the epigenetic pattern (Dai *et al*, 2020). Homozygous twins can be epigenetically indistinguishable during the early years of life, but with increased age, they exhibit remarkable differences in genomic distribution of epigenetics marks (Fraga *et al*, 2005). Hence, the epigenetic code while retaining past information provides a snapshot of the current status of a cell. Very recently, for example, and not free from debate, aging has been associated with loss of epigenetic information, rather than with cumulative mutations (Yang *et al*, 2023), indicating that the memory of DNA damage is impacting cell fate via epigenetic mechanisms beyond the ability of the cell to accurately repair DNA and prevent accumulation of mutations. Hence, the question is whether the epigenome can be used as mono‐dimensional unified record of the information produced by the convergency of GxE interactions, representing a quantitative biomarker to predict the risk of a given disease.

Epigenetic changes occur during the process of aging, and in particular, the status of methylation of cytosines included in the CpG islands in the genomic DNA has been properly recorded and classified as a score defining the “*epigenetic clock of ageing*” (Horvath & Raj, 2018). While epigenetic clocks display good predictability of the “*biological age*,” environmental exposure can influence epigenetic clocks resulting in processes that appear to accelerate epigenetic aging. Cigarette smoke largely influences epigenetic patterns of multiple organs and tissues; its influence on the methylation status of CpG islands impacts the epigenetic clock, indicating an acceleration of biological aging (Horvath & Raj, 2018). However, such “*acceleration of ageing*” is a proxy of increased disease risk, as a significant proportion of diseases are aging associated. This approach does not directly measure the risk of a disease and would not be naturally applicable to diseases that do not preferentially affect aged groups of individuals.

In this article, we discuss recent literature that supports the relevance of GxE for human health. We argue, however, that GxE should be conceived as a bidirectional, dynamic process that underlies interindividual variabilities and differential susceptibilities. While acknowledging the complexity of this multidimensional network, we point out the importance of deconvoluting the epigenetic code to develop quantitative measures of disease risk biomarkers (Fig 1B). We propose that the epigenetic code could represent the appropriate layer to investigate and decode the unified information generated by the convergence of GxE interactions (Fig 1C). To this end, we present a small collection of illustrative cases spanning various disease categories, wherein the significant role of GxE and the notable involvement of epigenetic regulations are distinctly evident. We provocatively introduce the term theoretical “epigenetic score meter” of disease to draw the attention of future research in delineation of epigenetic patterns associated with GxE. We propose that expanding the concept of epigenetic clock might help directly measure the risk level of specific diseases.

## Risk of disease: genes or environment? Unraveling the potential of epigenetics

### Asbestos toxicity and an epigenetic “suspect”

Asbestos toxicity has long been implicated in the development of malignant mesothelioma, a rare and deadly form of cancer affecting the linings of various organs. This disease serves as a prime example of an exposure‐driven condition, highlighting the significant occupational and environmental implications associated with asbestos exposure.

BRCA1‐associated protein 1 (BAP1) has emerged as a suspect in the context of asbestos‐related diseases. Over 200 families worldwide carrying germline mutations of BAP1 have been found to develop a condition known as “BAP1 cancer predisposition syndrome” (Carbone *et al*, 2013). Individuals with BAP1 mutations within these families have a remarkably higher likelihood of developing malignant mesothelioma, even with low exposure to asbestos or similar mineral fibers, compared to family members without the mutations exposed to similar environmental conditions. This observation suggests an interaction between genetic and environmental factors, supporting the notion of gene–environment (GxE) interaction in cancer susceptibility.

Intriguingly, BAP1 functions as a ubiquitin carboxy‐terminal hydrolase (UCH) and plays a role in histone posttranslational modifications by hydrolyzing ubiquitin at lysine 119 of histone 2A (H2AK119Ub). This activity is part of the polycomb repressive deubiquitinase complex (PR‐DUB), which is responsible for removing ubiquitin epigenetic marks from histones. The PR‐DUB complex interacts with polycomb repressive (PcG) proteins, which are known to regulate gene expression through histone modifications associated with transcriptional silencing (Carbone *et al*, 2013). Although the precise molecular mechanisms underlying BAP1's tumor‐suppressive function and its interaction with environmental agents remain largely unknown, it is believed that BAP1's activity is influenced by the complex interplay between genetic factors and environmental exposures, resulting in tissue‐specific epigenetic regulatory patterns. The case of BAP‐1 might be fully exploited to develop a proof of principle of epigenetic biomarkers of risk of cancer.

### Liver detoxification: genomic integrity and epigenetic regulations

Another relevant area lies in the detoxification processes related to aldehydes, which are intermediate products of endogenous reactions and are present in both food and environmental pollutants. Aldehydes can be detoxified by a family of dehydrogenases with varying tissue distribution and subcellular localization. One of these enzymes, mitochondrial aldehyde dehydrogenase 2 (ADH2), is responsible for converting toxic aldehydes, such as acetaldehyde, into harmless acetate. However, around 8% of the global population and 35–45% of the Asian population exhibit ADH2 deficiency due to specific genetic polymorphisms. These mutations significantly reduce ADH2's enzymatic activity and impair the ability to metabolize acetaldehyde effectively. Consequently, individuals with ADH2 deficiency are predisposed to various health conditions, including cardiovascular diseases, liver cirrhosis, and certain types of cancer (Garaycoechea *et al*, 2018).

Epigenetic mechanisms also play a role in aldehyde detoxification. Aberrant DNA hypermethylation can silence the ADH2 gene, leading to a loss of aldehyde detoxification capacity and increased genotoxicity. Moreover, the metabolic consequences of aldehyde detoxification directly impact the epigenetic methylation pattern (Garaycoechea *et al*, 2018). Formate, the detoxified product of formaldehyde, can enter the one‐carbon metabolism pathway and influence DNA and histone methylation processes. Imbalances in these processes can have far‐reaching consequences on the epigenetic integrity of cells.

### Genetics, epigenetics, and exposure in autoimmunity

Autoimmune disorders (ADs) generally have complex multifactorial etiologies, involving the combined contributions of various triggers and insults that can either initiate or influence the course of the autoimmune response. While genetic factors are often implicated in these conditions, which are sometimes polygenic in nature, understanding the associated exposome, or the totality of environmental exposures, presents a significant challenge.

A recently identified autoimmune disorder called vacuoles, E1 enzyme, X‐linked, autoinflammatory, and somatic (VEXAS) provides insight into the synergistic effects of genetic perturbations and stress responses leading to autoinflammation. The disease is characterized by hematological conditions, along with systemic infiltrations of neutrophils and macrophages in the skin, lungs, and blood vessels. A somatic mutation in codon 41 of the ubiquitin‐like modifier activating enzyme 1 (UBA1) gene has been identified as the cause, resulting in alterations to the open reading frame at codon 41. The interplay between the UBA1 gene mutation and the loss of function of the DNA demethylase TET2 has also been linked to myelodysplastic syndromes (Lotscher *et al*, 2021). Hence, epigenetic changes in the DNA methylome may contribute to the pathogenesis of these disorders while serving as potential biomarkers.

While our understanding of the contribution of the epigenome to VEXAS pathogenesis is still evolving due to its recent identification, more extensively studied autoimmune diseases provide evidence supporting the use of the epigenome as a biomarker or therapeutic target. For instance, single‐cell analyses of accessible chromatin have enabled the distinction of bone marrow‐derived myeloid cells (BMC) from microglia in an animal model of experimental autoimmune encephalomyelitis, offering insights into the activation signature of innate immune cells in autoimmune disorders like multiple sclerosis.

### Epigenetic interplays in autism spectrum disorder

Autism spectrum disorder (ASD), a neurodevelopmental condition characterized by a wide range of symptoms and impairments, remains an impossible puzzle due to its largely multifactorial nature. While there is evidence of heritability and the identification of various ASD‐associated mutations, the heterogeneous nature of these genetic variants limits their explanatory power, each accounting for a small fraction of cases (Gaugler *et al*, 2014). Thus, the surge in ASD prevalence from 1 in 10,000 in the 1970s to 1 in 36 in 2023 (source: CDC: cdc.gov/ncbddd/autism) cannot be solely attributed to genetics, changes in diagnostic practices, or increased awareness.

Genome‐wide association and large‐scale sequencing studies have successfully identified hundreds of ASD risk loci. Nevertheless, these genetic effects only account for approximately 59% of the etiological contribution to ASD, leaving a significant role for environmental factors (Gaugler *et al*, 2014). However, the underlying mechanisms by which these environmental factors impact ASD causation remain poorly understood.

Although limited, a growing body of research, primarily epidemiology based, has begun to explore gene–environment interactions (GxE) in autism. Notably, epigenetic processes have emerged as a crucial link between genetics and the environment in ASD. One intriguing susceptibility locus implicated in ASD development is CHD8 gene, which encodes a subunit of the SNF2H‐like ATP‐dependent chromatin remodeling factors, CHD (chromodomain helicase DNA binding). Alterations in CHD8 have been shown to directly influence epigenetic regulation and the transcription of genes involved in neuronal development and ASD (Wilkinson *et al*, 2015). The CHD8 susceptibility locus serves as a paradigmatic illustration of how epigenetic alterations and environmental exposures synergistically contribute to ASD etiology. This provides a compelling example of how changes in the epigenetic machinery, combined with environmental exposures, can imprint a distinct “record” in the epigenome, ultimately influencing disease risk.

In summary, while the genetic contributions to many diseases are well established, the complex interplay between genetics and environmental factors holds significant sway over their pathogenesis. Further exploration of gene–environment interactions, particularly through the lens of epigenetics, promises to shed light on the mechanisms underlying complex disorders and might pave the way to develop measurable risk assessment strategies.

## Probabilistic assessment of disease risk

The systematic evaluation of the risk factors of a given disease falls under the larger definition of disease risk assessment. The World Health Organization (WHO) invests substantial resources in the issuing of guidelines to minimize the “*behavioural risk factors*.” The estimation of risk factors mainly relies on the evaluation of behavioral, clinical, or familiar variables. This approach has large shortcomings due to the limitations of the models and the inability to predict uncertainties. For example, a reduction in alcohol intake, a low‐cholesterol diet, or an increase in daily physical activity are interventions from the category of “environmental factors,” but they are not tailored to the individual's genetics and personal history. Genetics has also been considered, but only indirectly. Family history is regarded as a relevant risk factor for many diseases, hence, demographic and family anamnesis combined with behavioral data have assisted the development of basic predictive tools of disease risk. However, these strategies have failed to accurately predict risk, as they work mainly with averaged population data and precaution principles. The approach to the problem requires a shift from generalized disease association studies to a proper quantitative risk assessment on the level of each individual.

A major limitation in the quantification of the risk of disease is related to the high degree of complexity of their etiologies. Only few diseases have a monogenetic origin, and even these are affected by genetic background and environmental factors. Even more complex is the situation for most pathologies that have multigenic contributions. Moreover, multiple environmental factors in combination with different timings can contribute to the pathogenesis of the disorder. The dissection of the GxE interaction can help (i) delineate a quantitative measure of the risk and (ii) better estimate the degree of uncertainty. Integration of multiple levels of regulations (i.e., genomics, transcriptomics, proteomics, etc.) has been suggested as strategy to delineate the intrinsic features of the biological system. This would also need to be integrated with the available information about behavior and environment of the individual. We suggest a strategy of simplification (reduction of dimensionality) by using epigenetic information as integrated readout for the consequence of GxE. Our idea assumes that all the relevant influences of genetics and environment are recoded in the epigenome, and this information can be decodified and correlated with a quantitative measure of a disease risk, as an “epigenetic score meter.” Although this appears provocative at present, there is precedence for the reproducible and quantitative decoding of extremely complex information, for instance, by the fast Fourier transformation in the 1960s, which allowed monitoring of nuclear weapons’ tests and is now an important basis of the science of earthquakes. Our postulate points out that the attention of future studies aiming at the deconvolution of GxE interactions should focus on the delineation of the epigenetic effects.

## Conclusions and perspectives

With a list of disease examples with very different etiology and molecular pathogenesis, we argue that virtually every individual disorder is the result of the combination of genetics and environmental components (toxicant, lifestyle, and pathogens). The degree of contribution of the two categories of “G” and “E” factors can vary largely, from very disproportioned cases of monogenic disorders, where the environment is possibly marginally influencing disease manifestation to more complex multifactorial pathologies. The unique combinations of each individual case generate a specific setting that accounts for interindividual variabilities. We further expanded this concept emphasizing the importance that epigenetic regulations can have in this intricate interplay. While the environment is a direct canonical trigger of epigenetic variations, genetics also exerts an influence on epigenetics. The results of the integration of the environmental signals in the epigenome can cause differential responses based on the specific genetic background.

We propose focusing the attention to the epigenome as a “read‐out” of GxE interactions; our idea, however, does not intend to belittle the importance of the delineation of genetic associations to disease susceptibilities (Fig 1). Characterization of loci of hypersusceptibilities represents a milestone in the deconvolution of GxE interaction. A systematic characterization has indeed to be further expanded also to the exposome, to determine the composition of the “E” associated with a disease. Evidence is emerging that compound mixtures must be considered in the process of risk assessment and experimental testing of chemicals.

Despite the substantial deployment of genomics technologies, epidemiological studies, and computational tools, there is still a gap in the ability to accurately predict disease risk in the population. While most of the studies have qualitative nature, there is a demand by public authorities, healthcare providers, and policymakers in having quantitative information on the risk probabilities. Here, we argue that a delineation of the epigenetic molecular basis can help provide the quantitative measure of how close an individual could be to the development of a disease. The determination of the best‐performing epigenetic marks such as “epigenetic score meter” remains an open area for future investigation. While DNA methylation well performs in prediction of the biological age, it might fail to fully integrate the information derived from GxE interactions, implying a need for delineation of more complex epigenetic read‐out, such as delineation of computational tools to define chromatin states. The fast development of technologies could, however, support the implementation of chromatin landscape studies in easily accessible human material, such as blood cells. Overall, while our postulation retains a theoretical nature at this stage, we would like to provocatively introduce the term “epigenetic score meter of disease” to point out the attention of future research in delineating the epigenetic basis of GxE interaction.

## Author contributions

**Alessio Butera:** Writing – original draft; project administration. **Lena Smirnova:** Writing – original draft. **Elisa Ferrando‐May:** Writing – original draft. **Thomas Hartung:** Conceptualization; writing – review and editing. **Thomas Brunner:** Conceptualization; writing – review and editing. **Marcel Leist:** Conceptualization; funding acquisition; writing – review and editing. **Ivano Amelio:** Conceptualization; supervision; funding acquisition; writing – original draft; writing – review and editing.

## Disclosure and competing interests statement

The authors declare that they have no conflict of interest.
